# Association between serum hormone levels in early pregnancy and risk of hypertensive diseases of pregnancy in women undergoing assisted reproduction

**DOI:** 10.1007/s10815-024-03212-8

**Published:** 2024-07-25

**Authors:** Rachel A. Martel, Victoria Lee, Abigail Armstrong, Maral Demirjian, Lorna Kwan, Zain A. Al-Safi

**Affiliations:** 1 Department of Obstetrics and Gynecology, Ronald Reagan UCLA Medical Center, Los Angeles, CA USA; 2 UCLA David Geffen School of Medicine, Los Angeles, CA USA; 3 Department of Urology, Ronald Reagan UCLA Medical Center, Los Angeles, CA USA; 4 Department of Obstetrics and Gynecology, UCLA David Geffen School of Medicine, 10833 Le Conte Avenue, 27-139 CHS, Los Angeles, CA 90095-1740 USA

**Keywords:** Preeclampsia, Gestational hypertension, Progesterone, Estradiol, Human chorionic gonadotropin

## Abstract

**Purpose:**

We examined the association between progesterone (P4), estradiol (E2), and human chorionic gonadotropin (hCG) levels in early pregnancy and the development of hypertensive diseases of pregnancy among women undergoing assisted reproduction.

**Methods:**

Retrospective study including patients who underwent frozen embryo transfer (FET), ovarian stimulation (OS), or unassisted conception (UC) and had a live singleton birth. The primary outcome was the development of hypertensive diseases of pregnancy (gestational hypertension, preeclampsia, HELLP, or eclampsia). Secondary outcomes were the development of fetal intrauterine growth restriction (IUGR), gestational diabetes mellitus, birth weight, and pre-term birth. Hormone levels and the development of the outcomes were correlated.

**Results:**

A total of 681 patients were included; 189 had FET, 193 had OS, and 299 had UC. Patients undergoing FET or OS were not more likely to develop hypertensive diseases of pregnancy compared with UC patients. While median levels of E2 and P4 were significantly different between P-FET and NC-FET patients (E2: 252 vs 317 pg/mL, P4: 64 vs 29 ng/mL, respectively; both *p* < 0.01), rates of hypertensive diseases of pregnancy did not significantly differ between those two groups. In the multivariate analyses, P4, E2, and hCG were not associated with the development of hypertensive diseases of pregnancy, but progesterone levels were significantly higher among those with IUGR. This remained consistent when the analysis was limited to FET patients.

**Conclusion:**

P4, E2, and hCG levels did not correlate with the development of hypertensive diseases of pregnancy but elevated progesterone levels did correlate with the development of IUGR.

**Supplementary Information:**

The online version contains supplementary material available at 10.1007/s10815-024-03212-8.

## Introduction

Preeclampsia (PE), a multi-system disease of pregnancy defined by the presence of new-onset hypertension with either proteinuria or other stigmata of end-organ damage at greater than 20 weeks of gestation [1], is one of the most important causes of both maternal and fetal morbidity and mortality worldwide [2, 3]. Hypertensive disorders of pregnancy account for 16% of pregnancy-related deaths, with PE specifically affecting 2–8% of pregnancies [4]. When not deadly, the disease can have significant consequences for multiple organ systems [4] leading to morbidities such as seizures, stroke, and renal failure [5]. Women who develop PE or other hypertensive disorders of pregnancy are twice as likely to develop chronic hypertension as well as cardiovascular and cerebrovascular diseases [6, 7]. In terms of fetal effects, PE is associated with preterm birth, fetal growth restriction, and perinatal death [1, 2].

Given the severity of PE, there has been significant research to identify at-risk women. Important risk factors for PE development include nulliparity, pre-gestational or gestational diabetes mellitus, chronic hypertension, renal disease, prior history of PE, multifetal gestation, obesity, and autoimmune disease [1, 8]. Both assisted reproductive technologies (ART) and advanced maternal age have also been implicated as risk factors [3, 8], an important consideration given that over 60% of women undergoing ART are older than 35 years [9]. Additionally, donated gametes are a risk factor for a maternal inflammatory response that increases the risk of PE [1].

While these risk factors have been established, the underlying pathophysiology of PE has not been clearly elucidated [5, 10]. As such, predicting who will ultimately develop the disease and initiating effective preventative interventions continues to be a major challenge in the field of obstetrics and gynecology [11]. Both the American College of Obstetrics and Gynecology and the National Institute for Health and Care Excellence have developed screening tool for preeclampsia; however, they identify as few as 2% and 34% of patients who go on to develop PE, respectively [3]. Developing more accurate screening tools, especially among women using ART, would facilitate timely prevention such as the initiation of aspirin prior to 16 weeks of gestation, an intervention that has been shown to decrease PE risk [12].

As hormonal support with estrogen and progesterone is a key component of several embryo transfer protocols, the goal of our study was to determine whether the hormonal milieu of early pregnancy can be predictive of PE risk. Therefore, we performed a retrospective study examining the association between progesterone, estradiol, and human chorionic gonadotropin (hCG) levels in early pregnancy and the development of PE and other hypertensive diseases of pregnancy among women undergoing ART.

## Methods

This was a retrospective cohort study performed at a single academic fertility center. All patients with a live birth who were seen for a fertility preconception consultation due to diagnosed infertility between 9/6/2013 and 12/17/2021 were screened for inclusion. Patients who conceived via tested or untested frozen embryo transfer (FET), ovarian stimulation (OS) with or without intrauterine insemination, and via unassisted conception (UC) were included. Patients were excluded if they had a multiple gestational pregnancy or if serum hormone levels and/or pregnancy outcomes were unavailable.

No hormonal supplementation was given to patients with OS or UC. Patients who underwent OS were started on either letrozole (2.5–7.5 mg per day) or clomiphene citrate (50–150 mg per day) on cycle day 3 for 5 days and monitored with ultrasound. When at least one follicle reached a size of > 18 mm, ovulation was triggered with recombinant HCG injection. Patients who underwent natural cycle frozen embryo transfer (NC-FET), defined as a spontaneous ovulatory monitored cycle with luteal phase support, were started on oral estradiol 2 mg three times daily and vaginal progesterone 100 mg three times daily 2 days after ovulation (as confirmed by a rise in serum progesterone), and FET was performed 5 days after ovulation. Hormone supplementation continued until 8 weeks of gestation. Patients who underwent programmed frozen embryo transfer (P-FET), defined as artificially preparing the endometrium with estrogen and progesterone, were started on oral estradiol 2 mg four times daily on day 2 of their cycle. After 12–15 days, depending on the patient’s response and endometrial thickness, daily intramuscular progesterone 100 mg in oil was initiated at the discretion of the patient’s provider. ET was scheduled on the sixth day of intramuscular progesterone. Hormone supplementation continued until 11 weeks of gestation. First hormone levels (estradiol, progesterone, and hCG) were drawn 9 days after blastocyst transfer or at approximately 4-weeks gestation for those who conceived via OS or UC. Second hCG levels were drawn 2 days after the first blood draw.

The primary outcome was the development of hypertensive disease of pregnancy, including gestational hypertension, PE, HELLP, or eclampsia, presented as a composite outcome. The criteria put forth by the American College of Obstetricians and Gynecologists were followed when diagnosing patients with all hypertensive diseases of pregnancy [13].

Secondary outcomes were the development of fetal intrauterine growth restriction (IUGR), defined by estimated fetal weight < 10th percentile for gestational age, development of gestational diabetes mellitus, birth weight, and pre-term birth (birth prior to 37-weeks gestation). Clinical outcomes were determined by the obstetrical team that evaluated patients throughout their pregnancy, at the time of delivery, and while admitted to the hospital postpartum. These outcomes were documented in the patient’s clinical record. Clinical records were then reviewed to determine whether study participants developed any of the primary or secondary outcomes. Institutional Review Board (IRB #21–000273) approval was obtained at the University of California at Los Angeles (UCLA).

Patient characteristics were compared between the FET, OS, and UC groups with chi-square (or Fisher’s exact) and Kruskal–Wallis tests. Hormone levels and pregnancy outcomes were also compared separately between each of the ART groups with the UC group with Wilcoxon rank-sum tests and chi-square (or Fisher’s exact) respectively. For this comparison, the FET group was further split into P-FET and NC-FET. A Hochberg and Bonferroni correction was applied to account for multiple pairwise tests. Finally, univariate analyses and multivariate logistic regressions, controlling for age, gravidity, BMI, chronic hypertension, and other underlying medical conditions that predispose to preeclampsia [13], history of gestational hypertension, and history of preeclampsia were conducted to evaluate whether hormone levels were associated with risk of developing hypertensive diseases of pregnancy and IUGR. A *p*-value less than 0.05 was considered statistically significant.

## Results

A total of 681 patients were included in the study. There were 189 patients who underwent FET (81 P-FET and 108 NC-FET). A total of 193 patients underwent OS, and 299 patients had a UC. The median age of study participants who achieved pregnancy after FET was 37 years compared to 35 years among those who achieved pregnancy after both OS and UC (*p* < 0.01). Parity differed between those undergoing FET, OS, and, UC with those undergoing UC more likely to be primiparous and those undergoing FET more likely to be multiparous (*p* = 0.03). Other baseline characteristics including median BMI, history of prior hypertensive disease of pregnancy, and history of chronic hypertension were not different between groups (Table 1).

**Table 1:** Baseline characteristics of study participants by type of conception: frozen embryo transfer, ovarian stimulation, and unassisted conception (*N* = 681)

	Embryo transfer (ET)	Ovarian stimulation (OS)	Unassisted conception (UC)	*p*-value
	*n* = 189	*n* = 193	*n* = 299	
Age, median (IQR)	37 (34, 39)	35 (32, 37)	35 (32, 37)	** < 0.01**^**a**^
Parity, % (*n*)				**0.02**
Primipara	59.8 (113)	65.3 (126)	71.6 (214)	
Multipara	40.2 (76)	34.7 (67)	28.4 (85)	
BMI, median (IQR)	22.6 (20.7, 24.9)	23.0 (20.7, 26.5)	23.0 (21.0, 26.0)	0.15^a^
Prior hypertensive disease of pregnancy, % yes (*n*)^b^	7.9 (8)	4.1 (4)	7.0 (11)	0.52
Chronic HTN, % yes (n)	4.2 (8)	4.7 (9)	3.3 (10)	0.75

Bold entries indicate statistical significance

^a^Kruskal-Wallis test

^b^356 patients had a prior pregnancy and were included in the percentage of patients with a prior diagnosis of a hypertensive disease of pregnancy

We assessed whether or not there were differences in hormone levels between those who developed gestational hypertension, preeclampsia without severe features, preeclampsia with severe features, superimposed preeclampsia, and early onset preeclampsia with a Kruskal–Wallis test. We found that there were no statistical differences in any of the hormones and concluded that a composite outcome of hypertensive diseases of pregnancy is appropriate (Supplemental Tables 1a and 1b). Pregnancy outcomes, including the development of hypertensive disease of pregnancy, did not differ significantly between those who conceived via FET or OS when compared with those who had a UC (Table 2), with the exception of a higher rate of IUGR among patients who underwent OS (8.8% vs 3.0%, *p* = 0.02) and a higher rate of low-birthweight infants among those who underwent P-FET (12.4% vs 3.7%, *p* = 0.03). Patients who underwent P-FET had significantly higher median progesterone levels than those who underwent UC (64.4 ng/mL vs 25.1 ng/mL, *p* < 0.01), as did patients who underwent OS (42.8 ng/mL vs 25.1 ng/mL, *p* < 0.01). When the analysis was restricted to those who underwent NC-FET or P-FET, estradiol and progesterone levels were significantly different between these two groups (Table 2). Median estradiol levels were lower (252 pg/mL vs 316.5 pg/mL, *p* < 0.01) and median progesterone levels were higher (64.4 ng/mL vs 28.8 ng/mL, *p* < 0.01) among those who underwent P-FET versus NC-FET. First and second hCG levels did not significantly differ by type of FET. Birth weight and rates of IUGR, hypertensive disease of pregnancy, and pre-term birth also did not significantly differ between these two sub-groups.

**Table 2:** Hormone levels and pregnancy outcomes in NC-FET (natural cycle frozen embryo transfer), P-FET (programmed frozen embryo transfer), and OS (ovulation stimulation) patients compared to patients with an unassisted conception (UC) (*N* = 681)

	NC-FET	P-FET	OS	UC	NC-FET vs UC	P-FET vs UC	OS vs UC	NC-FET vs P-FET
	*n* = 108	*n* = 81	*n* = 193	*n* = 299	*p*-value	*p*-value	*p*-value	*p*-value
Hormone levels, median (IQR)								
Estradiol, pg/mL	316.5 (254.0, 416.0)	252.0 (200.0, 307.0)	341.5 (207.5, 536.0)	240.0 (162.5, 360.5)	< 0.01^a^	0.99^a^	** < 0.01**^**a**^	** < 0.01**^**a**^
Progesterone, ng/mL	28.8 (18.3, 39.1)	64.4 (31.2, 85.0)	42.8 (29.7, 57.3)	25.1 (19.2, 31.6)	0.24^a^	** < 0.01**^**a**^	** < 0.01**^**a**^	** < 0.01**^**a**^
1st hCG, mIU/mL	171.0 (111.0, 278.5)	194.0 (133.0, 261.0)	251.0 (162.0, 408.0)	598.0 (216.0, 2174.0)	** < 0.01**^**a**^	** < 0.01**^**a**^	** < 0.01**^**a**^	0.96^a^
2nd hCG, mIU/mL	478.5 (257.0, 673.0)	435.0 (326.0, 649.0)	596.0 (379.0, 889.0)	797.0 (335.0, 1353.5)	** < 0.01**^**a**^	** < 0.01**^**a**^	0.09^a^	1.0^a^
Pregnancy outcomes, % (*n*)								
GDM	13.9 (15)	13.6 (11)	8.3 (16)	13.0 (39)	0.99	0.99	0.32	0.95
IUGR	5.6 (6)	8.6 (7)	8.8 (17)	3.0 (9)	0.69^b^	0.08^b^	**0.02**	0.41
Hypertensive disease of pregnancy	18.5 (20)	25.9 (21)	17.6 (34)	22.4 (67)	1.0	1.0	0.60	0.22
Preterm birth (GA < 37 weeks)	10.2 (11)	8.6 (7)	7.8 (15)	5.4 (16)	0.25	0.81^b^	0.84	0.72
Birth weight, g					0.29^b^	**0.02**^**b**^	0.26^b^	0.24
< 2500	8.6 (9)	12.4 (10)	6.8 (13)	3.7 (11)				
2500–3999	88.5 (92)	80.2 (65)	91.1 (174)	91.2 (271)				
≥ 4000	2.9 (3)	7.4 (6)	2.1 (4)	5.1 (15)				

Bold entries indicate statistical significance

*GDM*, gestational diabetes mellitus; *IUGR*, intrauterine growth restriction

^a^*p*-value corrected by Hochberg for the Wilcoxon rank-sum test

^b^*p*-value corrected by Bonferroni for Fisher’s exact test

There were no significant differences in estradiol, progesterone, or hCG levels between those who did and did not develop hypertensive disease of pregnancy (Table 3), even after controlling for age, gravidity, BMI, history of chronic hypertension, other underlying medical conditions that predispose to preeclampsia, history of gestational hypertension, and history of preeclampsia (supplemental Table 2a) and restricting the analysis to only patients who underwent P-FET or NC-FET (supplemental Table 2b and 2c). Outcomes did not change when patients with a history of chronic hypertension, other underlying medical conditions that predispose to preeclampsia, a history of gestational hypertension, and a history of preeclampsia were excluded from the analysis. Progesterone levels, however, were significantly higher among those who developed IUGR (44.2 ng/mL vs 30.3 ng/mL, *p* = 0.01) (Table 4). When the analysis was restricted to only patients with ART, progesterone levels were still significantly higher among those who developed IUGR (49.2 ng/mL vs 32.5 ng/mL, *p* = 0.04), and the first hCG level was significantly lower among those who developed IUGR (139 mIU/mL vs 193 mIU/mL, *p* = 0.05) (supplemental Table 3). Estradiol levels were not significantly higher in patients who developed IUGR than those who did not (Table 4). Among only patients who underwent P-FET or NC-FET, estradiol was not associated with IUGR (supplemental Table 3).

**Table 3:** Hormone levels in those who developed hypertensive diseases of pregnancy (gestational hypertension, PE, HELLP, or eclampsia) compared to those who did not develop hypertensive diseases of pregnancy among those who had programmed and natural cycle frozen embryo transfer, ovarian stimulation, and natural conception (*N* = 681)

	Hypertensive disease of pregnancy	No hypertensive disease of pregnancy	*p*-value^a^
	*n* = 143	*n* = 539	
Hormone levels, median (IQR)			
Estradiol, pg/mL	279.0 (197.0, 428.0)	286.0 (196.5, 392.0)	0.70
Progesterone, ng/mL	30.0 (21.4, 41.1)	30.9 (22.7, 48.1)	0.21
1st hCG, mIU/mL	241.5 (133.0, 557.0)	283.0 (153.0, 678.0)	0.22
2nd hCG, mIU/mL	473.5 (313.0, 846.0)	588.0 (340.0, 990.0)	0.09

^a^Wilcoxon rank-sum test

**Table 4:** Hormone levels in those who developed intrauterine growth restriction (IUGR) compared to those who did not develop IUGR among those who had programmed and natural cycle frozen embryo transfer, ovarian stimulation, and natural conception (*N* = 681)

	IUGR	No IUGR	*p*-value^a^
	*n* = 39	*n* = 642	
Hormone levels, median (IQR)			
Estradiol, pg/mL	308.0 (221.0, 533.0)	285.0 (196.0, 399.0)	0.12
Progesterone, ng/mL	44.2 (26.9, 59.2)	30.3 (22.1, 44.6)	**0.01**
1st hCG, mIU/mL	210.0 (114.0, 485.0)	278.5 (148.0, 660.0)	0.13
2nd hCG, mIU/mL	488.0 (303.0, 685.0)	578.0 (340.0, 964.0)	0.17

Bold entries indicate statistical significance

^a^Wilcoxon rank-sum test

## Discussion

Although PE is one of the most common diseases of pregnancy, its pathophysiology remains poorly understood. Some postulate that poor trophoblastic invasion impairs spiral artery remodeling leading to decreased placental blood flow and subsequent placental ischemia [14]. Prior to placental development, villous syncytiotrophoblasts and trophoblastic cells produce hCG which stimulates the corpus luteum to produce progesterone [15]. Disturbances in levels of hCG and progesterone may thus be markers of trophoblastic invasion and activity. The placenta also plays a key role in hormonal support of pregnancy. The luteal-placental shift occurs between 7 and 10 weeks [16], with the placenta responsible for estradiol and progesterone production after the shift takes place. Therefore, placental dysfunction may impact levels of estradiol and progesterone. Multiple studies have demonstrated an association between these hormone levels in the second and third trimesters and the development of PE [17–20]. However, research on hormonal associations with the development of PE in early pregnancy, especially among pregnancies that result from ART, has been limited. This is important given that hormonal support with vaginal or intramuscular progesterone is a key component of several embryo transfer protocols. Additionally, early markers of PE are particularly important as the predisposition to PE is likely established in the first trimester during trophoblast invasion, and aspirin prophylaxis is most effective when established between 12 and 16 weeks of gestation [12].

Recent studies have demonstrated an association between hormone levels in pregnancy and the development of PE, but the results are mixed. Aulitzky et al. studied 280 women who underwent in vitro fertilization (IVF) with a live birth and found that women whose first hCG level after embryo transfer was in the top quintile had a higher incidence of PE compared to those with a lower initial hCG level [21]. In contrast, an observational study of patients also undergoing IVF found that an hCG level < 50 on day 12 after embryo transfer was significantly associated with the risk of PE [22]. Berkane et al. explored the association between estradiol levels and the development of PE, finding that lower estradiol levels at the time of implantation are likely associated with PE. The authors hypothesize that estradiol is critical to normal trophoblast development and angiogenesis and that lower levels of estradiol thus lead to placental ischemia through impaired vessel formation and trophoblast invasion into the uterine decidua [23]. In contrast, Imudia et al. and Farhi et al. both concluded that an elevated estradiol level on the day of hCG trigger during IVF cycles with fresh embryo transfer is predictive of the development of PE and small for gestational age infants [24, 25]. They suggest, in direct contradiction to Berkane et al., that low levels of estradiol in early pregnancy are necessary to allow for normal spiral artery formation [24, 25]. 

Our retrospective study evaluated the relationship between early hormone levels in pregnancy and the risk of hypertensive diseases of pregnancy. In contrast to the above studies, our study did not find that hCG or estradiol levels correlated with the development of hypertensive diseases of pregnancy, even when the analysis was restricted to patients with FET. The discrepancy in results related to estradiol may be explained by the difference in the time of blood draw in each study. While our study examined estradiol levels at first blood draw after embryo transfer, Imudia et al. and Farhi et al. examined levels on day of hCG trigger in patients undergoing fresh embryo transfer [24, 25], and although they comment on the theoretical role of low estradiol in placental development, Berkane et al. ultimately drew their conclusion that low levels of estradiol are related to preeclampsia from data assessing estradiol levels in the third trimester of pregnancy and at delivery [23]. The inconsistency in results between previous studies when compared to each other and to our study and the difficulty in directly comparing studies that follow differing methodologic protocols (such as day of hormonal evaluation, or fresh versus frozen embryo transfer) highlight the need for further studies assessing the role of hCG and estradiol in early pregnancy.

Similarly, our study did not show an association between progesterone levels in the early first trimester and the development of hypertensive disorders of pregnancy, even when the analysis was restricted to patients with FET. Previous research has shown that progesterone level on the day of hCG trigger among women undergoing IVF is associated with risk of PE. However, this association did not persist when the analysis was confined to those who underwent frozen embryo transfer [26]. Luisi et al. evaluated the progesterone metabolite allopregnanolone which was shown to be elevated among women with hypertensive disorders of pregnancy in the second and third trimester [19]. Similarly, Salas et al. found that progesterone levels start to rise between 18 and 21 weeks in those who develop PE [20]. While the studies listed above show a relationship between increased progesterone in the second and third trimesters and PE and other hypertensive disorders, our study indicates that the relationship may not be established in the first trimester of pregnancy. However, further prospective studies are needed, especially given the known impact of progesterone on endometrial maturation, uterine angiogenesis, and vascular remodeling [27].

There is also a documented association between P-FET and an increased risk of hypertensive disorders of pregnancy when compared with NC-FET and UC [28]. A 2022 meta-analysis found a higher risk of hypertensive diseases of pregnancy among P-FET as compared to NC-FET patients but concluded that the evidence was low quality and not sufficient to make clinical recommendations [29]. This correlation was not seen in our study despite differences in hormone levels. The hormonal differences between P-FET and NC-FET are likely explained by the presence of a corpus luteum among patients undergoing NC-FET (leading to higher levels of estradiol) and the use of intramuscular rather than vaginal progesterone in the P-FET group (leading to higher levels of progesterone with P-FET) [30]. Our study suggests that these hormonal differences in early pregnancy may not be the mechanism behind the increased risk of developing hypertensive diseases of pregnancy. However, prior data has shown an increased risk for preeclampsia among IVF pregnancies without, as opposed to with, a corpus luteum [31]. Thus, other secretory products of the corpus luteum, such as relaxin [32, 33], may be more responsible for the discrepancies in rates of preeclampsia between P-FET and NC-FET patients that have been observed in other studies. These other components of the hormonal milieu of the luteal phase and early pregnancy are an important future direction in studying pregnancy outcomes among patients who undergo different FET protocols.

It should also be mentioned that patients undergoing FET in our study were more likely to be multiparous and older than those with UC or OI, both of which change their baseline risk of developing PE. However, FET patients in our study did not have a higher likelihood of developing PE or other hypertensive diseases of pregnancy than patients undergoing natural conception. Additionally, the comparison of hormone levels between those who did and did not develop hypertensive diseases of pregnancy was controlled for age and gravidity.

Multiple prior studies have shown an association between all three hormone levels in early pregnancy and rates of IUGR [24, 34–37]. Our study is consistent with previous data linking elevated progesterone after fresh embryo transfer with increased risk of IUGR [35] and consistent with a prior study linking lower hCG levels with risk of small for gestational age neonates (although the association found in this prior study was only significant in the late first trimester) [38]. Estradiol levels, however, were not consistently significantly associated with the risk of IUGR in our study. This is in contrast with studies demonstrating an increased risk of small for gestational age and low birth weight neonates among women undergoing IVF with fresh embryo transfer who had elevated estradiol levels on the day of hCG trigger [24, 36, 37]. As above, the timing of hormonal evaluation and the type of embryo transfer may explain the discrepant results.

Given our results showing elevated progesterone levels among those who develop IUGR and significantly higher levels of progesterone among P-FET compared to UC patients, it makes sense that P-FET patients had an elevated risk of neonates weighing < 2500 g compared to those with UC in our study despite no difference in rate of pre-term birth. However, when directly comparing P-FET and NC-FET patients, there was no difference in rates of IUGR or in the distribution of birthweights. It should additionally be noted that P-FET patients also had an increased percentage of neonates weighing > 4000 g compared to UC patients, although by a smaller margin than the increase in neonates weighing < 2500 g. It is possible that progesterone levels may have an impact on fetal weight at both extremes of growth. Our results support a relationship between progesterone levels and fetal/neonatal weight, but to accurately determine the relationship between those levels, type of FET, and risk of IUGR would require adequately powered prospective studies. Patients with OS similarly had significantly higher progesterone levels than those with UC and an increased risk of IUGR compared with UC. This points to continued evaluation of progesterone levels and risk of IUGR and neonatal weight as a fruitful avenue for continued investigation.

### Limitations and future directions

Our study has several limitations, chiefly its retrospective design. Additionally, hormonal measurements after OS and UC were drawn on an approximate rather than an exact date after conception, and variation in the day of draw between participants may have impacted results. The sample size of our study may have been underpowered to detect small differences between groups. Additionally, while prescribing aspirin in accordance with ACOG guidelines is standard of care at our center, information on which patients were taking it daily as prescribed and which patients initiated treatment at the recommended gestational age of 12 weeks was not available. Information on which embryos were tested for aneuploidy via PGT-A was also unavailable. The strengths of our study include that it was performed at a single center with consistent estrogen and progesterone supplementation and ART protocols. Additionally, the majority of patients were delivered at our center. Prospective studies are needed to further examine hormone level association in early pregnancy among frozen embryo transfer patients and their risk of developing PE.

## Conclusion

We did not find a relationship between progesterone, estradiol, or hCG levels in early pregnancy and the development of PE and other hypertensive diseases of pregnancy. This suggests that these hormones may be less important to the development of PE in early pregnancy than hormone levels after the placenta develops, which have been shown to correlate with PE risk. However, we did find a relationship between the development of IUGR and levels of progesterone and hCG, indicating that early pregnancy hormonal measurements remain important prognostic factors for the development of late pregnancy complications. Future research should investigate other early serum markers that could predict the development of hypertensive diseases of pregnancy.

## Supplementary Information

Below is the link to the electronic supplementary material.

Supplementary file1 (DOCX 28 KB)
