# Supplementary material for: Association between serum hormone levels in early pregnancy and risk of hypertensive diseases of pregnancy in women undergoing assisted reproduction
Source: J Assist Reprod Genet. 2024 Jul 25;41(9):2359–66. doi: 10.1007/s10815-024-03212-8 (PMC11405613; doi:10.1007/s10815-024-03212-8)
Supplement: Supplementary file 1 — Supplementary file1 (DOCX 28 KB) [file 10815_2024_3212_MOESM1_ESM.docx]

Supplementary information

Supplemental Table 1a. Hormone levels in those who developed gestational hypertension and different subtypes of preeclampsia. (n=143)

|  | Gestational hypertension | Preeclampsia without severe features | Preeclampsia with severe features | Superimposed preeclampsia without severe features | Superimposed preeclampsia with severe features | Early onset preeclampsia | p-value^a^ |
| --- | --- | --- | --- | --- | --- | --- | --- |
|  | n=65 | n=40 | n=22 | n=1 | n=11 | n=4 |  |
| Hormone levels, median (IQR) |  |  |  |  |  |  |  |
| Estradiol, pg/mL | 243.0 (183.5, 322.0) | 289.0 (190.0, 428.0) | 311.0 (260.0, 434.0) | 673.0 | 265.5 (252.0, 588.0) | 611.0 (418.5, 717.5) | 0.06 |
| Progesterone, ng/mL | 30.5 (22.5, 40.0) | 27.2 (18.7, 41.1) | 30.6 (22.2, 46.1) | 33.1 | 23.5 (14.0, 73.4) | 38.2 (28.1, 56.9) | 0.78 |
| 1^st^ hCG, mIU/mL | 253.0 (162.0, 596.0) | 228.0 (105.5, 568.5) | 180.5 (133.0, 547.0) | 290.0 | 275.0 (213.0, 2151.0) | 442.5 (308.5, 1904.5) | 0.61 |
| 2^nd^ hCG, mIU/mL | 572.5(344.5, 862.5) | 396.0 (260.0, 889.0) | 385.0 (331.0, 620.0) | 846.0 | 458.0 (326.0, 534.0) | 839.0 (537.0, 843.0) | 0.58 |
| ^a^ Kruskal-Wallis test | | | | | | | |

Supplemental Table 1b. Hormone levels in those who developed gestational hypertension and different types of preeclampsia among those who had programmed and natural cycle frozen embryo transfer (n=41)

|  | Gestational hypertension | Preeclampsia without severe features | Preeclampsia with severe features | Superimposed preeclampsia with severe features | Early onset preeclampsia | p-value^a^ |
| --- | --- | --- | --- | --- | --- | --- |
|  | n=15 | n=14 | n=8 | n=2 | n=2 |  |
| Hormone levels, median (IQR) |  |  |  |  |  |  |
| Estradiol, pg/mL | 284.0 (250.0, 420.0) | 280.5 (213.0, 436.0) | 304.5 (269.5, 382.0) | 712.5 (252.0, 1173.0) | 496.5 (304.0, 689.0) | 0.49 |
| Progesterone, ng/mL | 31.2 (21.4, 46.5) | 25.7 (16.9, 44.9) | 29.0 (19.6, 54.0) | 68.4 (16.8, 120.0) | 38.2 (32.5, 43.8) | 0.68 |
| 1^st^ hCG, mIU/mL | 192.0 (132.0, 253.0) | 188.0 (109.0, 261.0) | 147.0 (106.5, 182.5) | 192.0 (171.0, 213.0) | 329.0 (195.0, 463.0) | 0.58 |
| 2^nd^ hCG, mIU/mL | 437.0 (302.0, 664.0) | 388.0 (257.0, 634.0) | 341.5 (261.5, 409.0) | 385.5 (313.0, 458.0) | 688.0 (537.0, 839.0) | 0.38 |
| ^a^ Kruskal-Wallis test | | | | | | |

Supplemental Table 2a: Odds ratio for development of hypertensive disease of pregnancy (gestational hypertension, PE, HELLP, or eclampsia) for each hormone separately controlling for age, gravidity, BMI, history of chronic hypertension, other underlying medical conditions that predispose to preeclampsia, history of gestational hypertension, and history of preeclampsia (N=681).

|  | Estradiol | Progesterone | 1st hCG | 2nd hCG |
| --- | --- | --- | --- | --- |
|  | aOR (95% CI) | aOR (95% CI) | aOR (95% CI) | aOR (95% CI) |
| Hormone | 1.3 (0.8, 2.2) | 1.2 (0.7,1.8) | 1.1 (0.7, 1.8) | 0.9 (0.5, 1.5) |
| Age | 1.0 (0.9, 1.0) | 1.0 (0.9, 1.0) | 1.0 (0.9, 1.0) | 1.0 (0.9, 1.1) |
| Gravidity (0 as referent) |  |  |  |  |
| 1 | 0.7 (0.4, 1.3) | 0.8 (0.5, 1.3) | 0.9 (0.6, 1.5) | 0.9 (0.5, 1.5) |
| 2 | 1.0 (0.6, 1.6) | 0.8 (0.5, 1.2) | 0.9 (0.5, 1.4) | 1.0 (0.6, 1.6) |
| BMI (normal as referent) |  |  |  |  |
| Overweight | 1.5 (0.9, 2.6) | 1.3 (0.8, 2.2) | 1.5 (1.0, 2.5) | 1.5 (0.9, 2.6) |
| Obese | **2.4 (1.2, 4.6)** | **2.8 (1.6, 5.0)** | **3.0 (1.7, 5.1)** | **2.5 (1.4, 4.5)** |
| Chronic Hypertension | 2.7 (1.0, 7.4) | **2.6 (1.1, 6.0)** | **2.7 (1.2, 6.0)** | 1.7 (0.7, 4.3) |
| Underlying Medical Conditions | 1.5 (0.4, 4.9) | 1.5 (0.5, 4.9) | 1.4 (0.4, 4.3) | 1.6 (0.5, 5.0) |
| Prior Gestational HTN | 2.9 (0.5, 17.5) | 3.1 (0.7, 13.8) | 2.5 (0.6, 10.5) | 0.8 (0.1, 6.7) |
| Prior Preeclampsia | 1.4 (0.4, 5.4) | 1.2 (0.3, 4.7) | 1.1 (0.3, 4.3) | 0.8 (0.2, 3.9) |

Supplemental Table 2b: Hormone levels in those who developed hypertensive diseases of pregnancy (gestational hypertension, PE, HELLP, or eclampsia) compared to those who did not develop a hypertensive disease of pregnancy among those who had programmed and natural cycle frozen embryo transfer (n=189)

|  | Hypertensive Disease of Pregnancy | No Hypertensive Disease of Pregnancy | p-value^a^ |
| --- | --- | --- | --- |
|  | n=41 | n=148 |  |
| Hormone levels, median (IQR) |  |  |  |
| Estradiol level, pg/mL | 284.0 (250.0, 420.0) | 289.0 (220.0, 353.0) | 0.37 |
| Progesterone level, ng/mL | 31.2 (21.4, 46.5) | 34.0 (23.5, 55.0) | 0.52 |
| 1^st^ hCG level, IU/mL | 192.0 (132.0, 253.0) | 186.5 (114.0, 274.5) | 0.98 |
| 2^nd^ hCG level, IU/mL | 437.0 (302.0, 664.0) | 474.0 (305.0, 660.0) | 0.80 |
| ^a^ Wilcoxon rank-sum test | | | |

Supplemental Table 2c: Odds ratio for development of hypertensive disease of pregnancy (gestational hypertension, PE, HELLP, or eclampsia) for each hormone separately among those who had programmed and natural cycle frozen embryo transfer controlling for age, gravidity, BMI, history of chronic hypertension, other underlying medical conditions that predispose to preeclampsia, history of gestational hypertension, and history of preeclampsia (n=189)

|  | Estradiol | Progesterone | 1st hCG | 2nd hCG |
| --- | --- | --- | --- | --- |
|  | aOR (95% CI) | aOR (95% CI) | aOR (95% CI) | aOR (95% CI) |
| Hormone | 1.8 (0.8, 4.1) | 1.7 (0.7, 3.9) | 0.8 (0.3, 1.9) | 0.9 (0.4, 2.2) |
| Age | 0.9 (0.8, 1.0) | 0.9 (0.8, 1.0) | 0.9 (0.8, 1.0) | 0.9 (0.8, 1.0) |
| Gravidity (0 as referent) |  |  |  |  |
| 1 | 0.7 (0.4, 1.3) | 0.5 (0.2, 1.3) | 0.5 (0.2, 1.3) | 0.5 (0.2, 1.4) |
| 2 | 1.0 (0.6, 1.6) | 0.7 (0.3, 1.6) | 0.7 (0.3, 1.7) | 0.7 (0.3, 1.7) |
| BMI (normal as referent) |  |  |  |  |
| Overweight | 0.7 (0.2, 2.0) | 0.6 (0.2, 1.9) | 0.7 (0.2, 2.0) | 0.7 (0.2, 2.0) |
| Obese | 3.3 (0.8, 12.7) | **3.5 (1.0, 12.5)** | **4.1 (1.1, 14.5)** | **3.9 (1.1, 13.8)** |
| Chronic Hypertension | 1.1 (0.1, 9.9) | 0.7 (0.1, 6.3) | 0.9 (0.1, 7.5) | 0.9 (0.1, 7.3) |
| Underlying Medical Conditions | 2.0 (0.3, 14.2) | 2.4 (0.3, 15.9) | 2.0 (0.3, 14.0) | 2.1 (0.3, 14.2) |
| Prior Preeclampsia | 1.3 (0.1, 14.5) | 0.9 (0.1, 10.8) | 1.1 (0.1, 12.1) | 1.0 (0.1, 11.3) |

Supplemental Table 3: Hormone levels in those who developed IUGR compared to those who did not among those who had programmed and natural cycle frozen embryo transfer (n=189)

|  | IUGR | No IUGR | p-value^a^ |
| --- | --- | --- | --- |
|  | n=13 | n=176 |  |
| Hormone levels, median (IQR) |  |  |  |
| Estradiol level, pg/mL | 256.0 (183.0, 394.0) | 289.0 (228.0, 362.0) | 0.53 |
| Progesterone level, ng/mL | 49.2 (30.2, 85.0) | 32.5 (22.5, 50.3) | **0.04** |
| 1^st^ hCG level, IU/mL | 139.0 (81.0, 204.0) | 193.0 (130.5, 276.0) | **0.049** |
| 2^nd^ hCG level, IU/mL | 342.0 (169.0, 458.0) | 475.0 (306.0, 673.0) | 0.054 |
| ^a^ Wilcoxon rank-sum test | | | |
